# Light Therapy in Post-Traumatic Stress Disorder: A Systematic Review of Interventional Studies

**DOI:** 10.3390/jcm13133926

**Published:** 2024-07-04

**Authors:** Florian Millot, Francky Teddy Endomba, Nathalie Forestier

**Affiliations:** 1 Psychiatry Internship Program, University of Burgundy, Dijon, France; 2 Service de Psychiatrie Adultes, Centre Hospitalier Universitaire, Dijon, France; 3 INSERM LNC UMR1231, University of Burgundy, Dijon, France

**Keywords:** light therapy, post-traumatic stress disorder, PTSD, systematic review, interventional studies

## Abstract

**Background**: Due to limitations in treatment strategies for post-traumatic stress disorders (PTSD), therapeutic options such as light therapy (LT) have garnered some interest in recent years. We aimed to review the effectiveness of LT in patients with PTSD. **Methods**: Using PubMed, PsycINFO, Web of Science, the Cochrane database, ClinicalTrials.gov, and PTSDpubs, we systematically searched for papers assessing the effect of LT in PTSD. We evaluated the risk of bias of included studies using the Cochrane handbook, and synthesized our findings following the Preferred Reporting Items for Systematic reviews and Meta-Analyses guidelines (PRISMA 2020). **Results**: From 140 initial papers, we included four randomized controlled trials (RCTs) and one single-arm study. The study sample size ranged between 15 and 82, the mean age (standard deviation) varied between 31.4 (8.8) and 44.9 (11.8) years, and LT was applied for four or six weeks. The risk of bias was low in three studies, and of some concern in the two other trials. Most studies reported no significant differences between LT and placebo regarding effects on subjective (sleep quality and insomnia severity) and objective sleep parameters. LT was associated with a significant improvement in PTSD symptom severity in the single-arm study and two RCTs, as well as a greater retention of extinction learning. Results on depression and anxiety were discrepant. **Conclusions**: This review revealed that relevant studies are scarce, with promising findings concerning PTSD symptoms, but inconsistencies for the other parameters. Further research projects are needed to better explore this topic.

## 1. Introduction

### 1.1. Rationale

Post-traumatic stress disorder (PTSD) is a mental health illness that may arise following exposure to a traumatic event, and concerned individuals can experience a wide range of long-lasting symptoms as well as an alteration in quality of life [1,2]. These symptoms can be grouped into intrusion symptoms (including traumatic nightmares), persistent avoidance, negative alterations in cognitions and mood, as well as alterations in arousal and reactivity (with an impact on sleep–wake cycles) [1]. PTSD can affect direct victims of traumatic or frightening events (such as war situations, serious accidents, natural disasters, sexual violence, terrorist act, military combat, and physical or emotional abuse), but also witnesses [1,2,3]. Exposure can be indirect when a traumatic event is experienced by someone else who then shares their experience with others, and can be repeated/prolonged in some cases [1,2,3]. The lifetime prevalence of PTSD varies according to socio-demographic patterns, with, for instance, differences between military and civilian populations, and countries experiencing war or not [2,3]. It is estimated that PTSD occurs in 5-10% of the population [2,3], and the point prevalence of PTSD was estimated at 26.5% in a meta-analytic review that targeted countries affected by war between 1989 and 2019 [4]. The risk, rate, and severity of PTSD seem to be higher in women compared to men, with a potential role of non-biological sex-specific factors and biological determinants [5,6]. These biological factors are related to fear systems which include GABAergic, glutamatergic, and cholinergic signalling, as well as specified brain circuits [5,6,7]. The neural circuits of fear encompass the prefrontal cortex, the hippocampus, and the amygdala, with respective roles in fear learning, contextual fear expression, and fear memory formation/inhibition [2,3,5]. The greater frequency of PTSD in women can also be explained by a greater exposure to traumatic experiences [2,6]. There also seem to be age-related differences in acute stress and PTSD symptoms, with lower rates in older age groups [7,8]. In more than 50% of cases, PTSD is associated with mood, anxiety, substance-use disorders, suicidal behaviours, and sleep disturbances [2,3,9]. More specifically concerning sleep, the pooled prevalence of insomnia in PTSD/PTSS (post-traumatic stress symptoms) has been recently estimated at 63%, with a significant correlation between these two disorders [10].

The currently used treatments for PTSD include pharmacological agents (such as selective serotonin reuptake inhibitors and blockers of α-adrenergic receptors) and psychological interventions, notably including trauma-focused interventions (such as trauma-focused cognitive behavioural therapy protocols, eye movement desensitization and reprocessing, and narrative exposure therapy) as well as non-trauma-focused psychotherapies (such as supportive therapy, mindfulness and present-centred therapy, and interpersonal therapy) [2,3,11,12]. Gender-based differences in outcomes have been reported following trauma-focused psychological interventions for PTSD [13]. However, there are limitations pertaining to the access and implementation of psychotherapies, partially due to the lack of trained professionals, especially in low- and middle-income countries [12,14]. Also, there are significant inconsistencies across PTSD patients regarding responses to available treatments. Indeed, for a certain number of patients, the therapies currently used for PTSD attenuate the symptoms of the disease, but there is no remission [2,11]. Persistent symptoms after treatments notably include insomnia (48 to 63%) and nightmares (13 to 45%), with frequency variations depending on studies, populations, and treatments [15,16,17,18]. Residual symptoms also emerged for depression, anxiety, and altered quality of life [19]. Considering this context, there is growing interest in the development of new therapeutic options for PTSD. In particular, light therapy (LT) has been the subject of some interest in recent years.

Light therapy (LT), also known as bright light therapy (BLT), is a treatment based on the effect of light on various psychophysiological spheres including notably mood and sleep–wake cycles. More specifically, blue light acts on the intrinsically photosensitive retinal ganglion cells (ipRGCs), projecting to the suprachiasmatic nucleus (SCN) in the hypothalamus for circadian rhythm regulation, and on mood-regulating areas [20,21,22]. Light is also reported to potentially act on brain structures/regions such as cingulate regions, the hippocampus, and the amygdala, which are implicated in the pathophysiology of PTSD according to various literature reports [20,22,23,24]. Light is known to influence sleep through homeostatic and circadian processes, with biological clock synchronisation and circadian phase shifting [20,25,26]. Interestingly, LT has been found to be effective in the treatment of circadian rhythm disorders and insomnia, with a recently published meta-analysis showing some effectiveness for sleep maintenance in insomnia disorders [25,26]. Sleep is reported to play an important role in memory consolidation, and sleep alterations (especially sleep deprivation) can impair fear memory processes [27,28]. By acting on sleep, LT could thereby influence these processes, which are involved in PTSD. BLT is recognized to be an effective treatment for seasonal affective disorder (SAD) [29], with data demonstrating efficacy in unipolar and bipolar depression without a seasonal pattern [30]. BLT is thus a valuable candidate among the therapeutic inventory available for the treatment of nonseasonal depression, as an adjuvant therapy (augmentation strategy) to antidepressant medication [30,31]. Notably due to its documented action on mood and sleep, which are frequently altered in individuals with PTSD, previous studies have assessed the effects of LT in these patients [32,33].

The epidemiologic burden of PTSD, notably through comorbidities and residual symptoms related to sleep and mood, the limitations of current recommended therapies, the recent interest in light therapy, and the lack of summarizing data, led us to perform this review.

### 1.2. Objectives

This systematic review aimed to assess the efficacy and safety of LT in people with PTSD, and our report is guided by the Preferred Reporting Items for Systematic reviews and Meta-Analyses (PRISMA 2020) statement [34]. The PRISMA checklist is available in Supplementary Table S1.

## 2. Methods

### 2.1. Eligibility Criteria

We targeted studies published from inception until 4 March 2024, with an interventional design. This included single-arm, randomized, and non-randomized trials. The other inclusion criteria were based on the PICO (population, intervention, comparator, and outcome) approach (Supplementary Table S2) which is indicated to build research questions dedicated to effectiveness reviews [35]. More specifically, the targeted study population was patients with PTSD, the considered intervention was light therapy, the comparator was the placebo light condition (in randomized studies), and the targeted outcomes were cognitive-behavioural features (including sleep and PTSD symptoms), affective parameters, as well as neuroanatomical and neurofunctional factors. We excluded studies performed on humans under 18 years old, research on animal models, case reports, letters to the editor, comments, duplicates, articles for which the full text was not available even after request, the least recent studies if several studies were based on the same population, and studies written in a language other than English or French. Our eligibility criteria are displayed in Table 1.

### 2.2. Information Sources and Search Strategy

We systematically searched for relevant articles using six digital databases, including PubMed, PsycINFO, Web of Science, the Cochrane database, ClinicalTrials.gov, and PTSDpubs.

To retrieve relevant papers, we built dedicated research queries by joining terms related to PTSD and light therapy. For illustration, the search strategy applied to PubMed was as follows: (“Posttraumatic stress” OR “Post traumatic stress” OR “Post-traumatic stress” OR “Psychotrauma*” OR “Trauma-related” OR “Trauma related” OR PTSD) AND (“Blue-light” OR “Blue light” OR “Laser light” OR “Laser-light” OR “Bright light therap*” OR “Morning light” OR “Light-therap*” OR “Light therap*” OR “Light treatment*” OR “Phototherap*” OR “Photo therap*”). The search strategies pertaining to the other databases are displayed in Supplementary Table S3.

### 2.3. Selection Process

After applying the search strategy, we performed a two-step selection process. The first step was based on the relevance of titles and/or abstracts, and the second step was based on full-text retrieval and appraisal with respect to the general objective of the review. Two authors were implicated in this process (FM and FTE), and disagreements were resolved through consensual decisions or the intervention of the third investigator (NF). We contacted authors of articles for which the full-text was not available, and a lack of response within one month led to the exclusion of the concerned paper. The reasons for exclusion are reported in a dedicated flow chart.

### 2.4. Data Extraction and Collected Data

From each selected paper, we extracted bibliometric information (first author’s name and year of publication), as well as information regarding the location, the period, the population (with the tool used to diagnose/screen PTSD), interventions (technical features of LT and control conditions), the gender ratio, and the age of participants. We also collected data on assessed outcomes, especially sleep parameters, anxiety, mood features, PTSD symptoms/severity, neuropsychological features, neuroanatomical data, and safety outcomes. Two authors were involved in this step (FM and FTE), and discrepancies were handled with the collaboration of the third author (NF).

### 2.5. Study Risk of Bias Assessment

For randomized controlled trials, the risk of bias assessment was performed with the Cochrane Risk of Bias (RoB) 2.0 tool, a tool made of five domains identified as potential sources of bias [36]. These domains include risk of bias resulting from the randomization process, risk of bias resulting from deviations from planned interventions, missing data on the outcome, risk of bias due to measurement of the outcome, and risk of bias in the selection of reported results. From the assessments of each domain, we were able to conclude on the overall risk of bias for each study. For non-randomized studies, we used the Risk of Bias in Non-randomized Studies—of Interventions (ROBINS-I) assessment tool [37]. This tool addresses biases due to confounding, to participant selection, to intervention classification, to deviations from intended interventions, to missing data, to outcome measurement, and to the reporting of results. For one-arm trials (uncontrolled clinical trials), we used the bias domains pertaining to the risk of bias in uncontrolled before–after studies, as stated in the Cochrane handbook [37]. These domains include bias due to confounding, bias in selection of participants into the study, bias in classification of interventions, bias due to deviations from intended interventions, bias due to missing data, bias in measurement of outcome, and bias in selection of the reported result. In case of divergences between risk of bias assessors (FM and FTE), the decision was made using a third opinion (NF).

### 2.6. Synthesis Methods

To summarize our findings, we used tables, figures, and narrative syntheses. More specifically, we used a PRISMA-based flow chart to illustrate the selection process, a tabular report to display individual characteristics of included studies, as well as data pertaining to the risk of bias assessment. We used tabular and text syntheses to report results related to efficacy and safety outcomes.

## 3. Results

### 3.1. Study Selection

**Figure 1:**
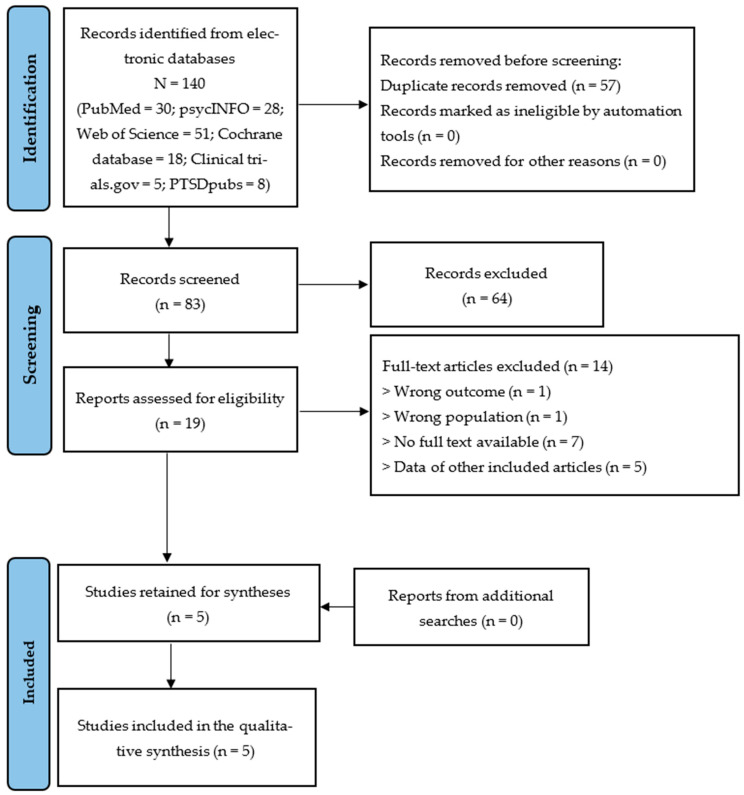
Flow chart of study selection.

After applying the search strategy on the selected databases, we obtained 140 papers from which 57 duplicates were removed and 19 full-texts were searched/assessed for eligibility. From these articles, 14 were excluded due the unavailability of full texts (*n* = 7), the duplicated pattern of data (*n* = 5), the wrong outcome (*n* = 1), and the wrong population (*n* = 1). We finally included five articles for our review [32,33,38,39,40], and the selection process is summarized in Figure 1.

### 3.2. Study Characteristics

All our eligible studies took place in the United States of America. Four studies were randomized controlled trials, and the last was an uncontrolled clinical trial (open-label, single-arm study) [32]. The assessment of PTSD was based on the DSM-5 in four studies, and the DSM-IV in one study [39]. The study sample size ranged between 15 and 82, and the mean age (reported in three studies) varied between 31.4 (8.8) years and 44.9 (11.8) years. Regarding the intervention of interest, the period of light therapy was 4 weeks in three studies [32,39,40], and 6 weeks for the other trials [33,38], with a morning exposure of 30 min [33,38] to one hour [32,39,40]. The light intensities (active group) were 10000 lux in two studies [32,39], 214 lux in two studies [33,38], and 500 lux in the last study [40]. The general features of our included studies are detailed in Table 2.

The most frequently assessed outcomes in the studies included in our review were sleep parameters, PTSD symptoms/severity, depressive symptoms, and anxiety. Regarding sleep appraisal, we had questionnaires such as the Epworth Sleepiness Scale (ESS), the Pittsburgh Sleep Quality Index (PSQI), the Insomnia Severity Index (ISI), and the Disturbing Dreams and Nightmare Severity Index (DDNSI), as well as actigraphy measurements such as total sleep time (TST), wake after sleep onset (WASO), and sleep efficiency (SE). More specifically, the PSQI is a self-rated questionnaire which assesses sleep quality and disturbances over a 1-month time interval [41], and the ISI is a brief tool developed to assess the nighttime and daytime severity of insomnia [42]. The ESS is a questionnaire widely used to evaluate daytime sleepiness [43], and the DDNSI is an instrument conceived for the assessment of the frequency of nightmares, as well as their related distress [44]. PTSD symptoms/severity were assessed through the Clinician-Administered PTSD Scale for DSM-5 (CAPS-5), and the Post-traumatic Stress Disorder Checklist for DSM-5 (PCL-5). The CAPS-5 is a structured diagnostic interview that address the frequency and severity of each PTSD symptom [45], while the PCL-5 is a self-rated measure of DSM-5 PTSD symptoms [46]. Depressive symptoms were assessed through the Hamilton Depression Scale, 9-item Patient Health Questionnaire (PHQ-9), and Beck Depression Inventory (BDI), while anxiety was appraised using self-reported scales of anxiety state.

As another type of outcome, Killgore et al. collected structural magnetic resonance imaging (MRI) data at 3T, and anatomical data were acquired with a high-resolution T1-weighted 3D magnetization prepared rapid acquisition gradient echo (MPRAGE) sequence [33]. T1 weighted structural images were pre-processed, and processed grey matter volume (GMV) data were analysed [33]. Vanuk and colleagues appraised skin conductance response during fear conditioning (difference between the “extinguished” and the “never-extinguished” stimuli at follow-up), as well as brain activation through functional MRI (exposure to a previously conditioned stimuli in a novel context) [38].

### 3.3. Risk of Bias in Studies

The risk of bias was found to be low for two studies [33,40], and of some concern for the two other RCTs. For these two last studies, the potential source of bias were deviations from the intended interventions/effect of assignment to intervention. Table 3 shows the assessment of the methodological quality of the RCTs (*n* = 4) included in our study. Since the study from Elliot et al. was an open-arm trial, we assessed its methodological quality based on the Cochrane handbook section pertaining to the risk of bias in uncontrolled before–after studies, and the overall risk was low.

### 3.4. Results of Syntheses

Most of our included studies (*n* = 4) found no significant effect of light therapy on subjective sleep parameters, notably sleep quality (assessed through the PSQI), insomnia severity (appraised with the ISI), and disturbed dreams/nightmares (evaluated through the DDNSI) [33,38,39,40]. On the other hand, the open-label single-arm trial assessing the effect of LT on veterans with a history of traumatic brain injury (TBI) found that subjects with comorbid PTSD had 5.76 times the odds (*p* = 0.041) of showing a response to light therapy (a 4-point decrease in ISI) compared to subjects without PTSD [32]. Also, veterans with a history of TBI and comorbid PTSD had higher percentages of post-intervention change [32]. Regarding objective sleep parameters (obtained through actigraphy), Killgore and colleagues found that LT led to significant increases in total time in bed and total sleep time compared to the placebo condition [33]. This was not the case for WASO and SE, in both per-protocol and intention-to-treat analyses [33]. Youngstedt et al. reported no significant differences in actigraphy parameters, notably TST, WASO, and SE [39]. Zalta et al. had similar results, with a non-significant improvement in wake time, TST, and WASO with the active LT [40].

Concerning PTSD severity, one study reported a significant improvement of dedicated scores with LT when compared to the placebo condition [39]. In this study, LT also produced a significantly greater proportion of treatment response for the CAPS (Clinician-Administered PTSD Scale for DSM-5) and PCL-M [39]. Zalta and colleagues found that the probability of reaching minimal clinically relevant modifications in PTSD and depression symptoms was higher in patients with active LT [40], while changes in depression and anxiety did not differ between treatments in the study of Youngstedt et al. [39]. In the study of Vanuk et al. when viewing previously feared and then extinguished stimuli, BLT participants showed reduced responses in brain regions implicated in anxiety [38]. In the same study, both groups (active and placebo) improved in PTSD symptoms and severity, assessed through the CAPS-5 [38]. Killgore et al. displayed a significantly greater GMV within the left amygdala in participants that received BLT (compared to ALT), but the expected augmentation in GMV within the medial prefrontal cortex was not found [33]. The left amygdala GMV increase was correlated with improvements in sleep quality (PSQI scores reductions over 6 weeks of treatment) and nightmare severity (declines in DDNSI scores over the treatment period) [33]. Vanuk et al. reported a better consolidation of extinction memories with LT, through an improvement of sleep parameters [38]. The LT intervention was well tolerated by patients, as reported by three of the selected studies [32,39,40]. Table 4 details information pertaining to the outcomes of interest in each included study.

## 4. Discussion

We conducted this systematic review of interventional studies in order to assess the effectiveness of light therapy in PTSD. In summary, we found that LT had a positive effect on sleep parameters, but without differences when compared to the placebo condition. However, compared to the placebo condition, BLT was associated with a significant improvement in PTSD severity symptoms, a significant increase in GMV in the left amygdala, and a greater retention of extinction learning. There were inconsistences regarding comparative results on depression and anxiety symptoms. The overall risk of bias was low for three studies, and of some concern in the two other trials.

### 4.1. General Interpretation in the Context of Other Evidence

The relevant effect of LT on PTSD symptoms might be explained by the implication of shared brain circuits/areas. Of note, the neural systems implicated in PTSD pathophysiology include fear learning and memory, threat detection, executive function, emotion regulation, and contextual processing [2,3,47]. More specifically, fear learning abnormalities (with persistence of fear responses in patients with PTSD) involves the amygdala, while threat detection and salience are related to the amygdala, the dorsal anterior cingulate cortex (ACC), and the insula [2,3,47,48]. Fronto-parietal regions (dorsolateral prefrontal cortex and ventrolateral prefrontal cortex) are implicated in executive function and emotion regulation, and contextual information processing (which is altered in PTSD) depends on the medial prefrontal cortex and the hippocampus [2,3,48]. There are reports suggesting that BLT has significant effects on memory, especially through an action on the hippocampus [22,24]. In addition, as stated by the study of Vanuk et al., BLT might also positively influence memory circuits by improving sleep quality [38]. Indeed, sleep plays a key role in fear memory consolidation, and PTSD is characterized by abnormalities in fear learning mechanisms [3,28,49]. On this point, Feng and colleagues reported that sleep deprivation may interact with the amygdala control through the ventromedial prefrontal cortex (vmPFC), and increase arousal signalling in the amygdala–insula pathway during fear consolidation [49]. LT seem also to act on visual circuits related to the lateral habenula, a structure regulating the connection between the limbic system and monoaminergic centres in the midbrain [20,22,24]. LT has been associated with an increase in the regional cerebral blood flow (rCBF) in the frontal and cingulate regions as well as the thalamus, especially in patients with SAD [24,50]. In addition, some evidence suggests that morning light may improve traumatic stress by influencing (reducing) amygdala reactivity [23,51]. In our review, one study found an increase in left amygdala volume after BLT [33], and other published data reported greater positive connectivity between the right amygdala and a region within the left dorsolateral prefrontal cortex in individuals who received blue wavelength light [23]. Killgore and colleagues reported that improvements in sleep quality and nightmare severity were correlated with augmentations in left amygdala volume [33]. In one of the review’s included studies, BLT resulted in a significant reduction in activation responses within the left insular cortex [38]. Based on some literature findings, inflammation and brain plasticity (through neurotrophic factor regulation) are involved in PTSD and more specifically in PTSD-related sleep disturbances [2,52,53], and BLT seems to have an effect on neuroinflammatory markers as well as neuroplasticity markers, especially in corticolimbic regions [50]. According to a study that assessed plasma brain-derived neurotrophic factor (BDNF) levels before and after BLT in treatment-resistant depression, responders and remitters had higher post-treatment plasma BDNF concentrations than patients who did not achieve response or remission [54]. Elliott and colleagues, while using morning BLT in veterans with traumatic brain injury, found improvements in IL-6 and TNF-ɑ, peripheral pro-inflammatory cytokines, and trends toward potential improvements in markers of neuronal injury and neuroinflammation [32]. Other points to consider are the key role of the hypothalamic–pituitary–adrenal (HPA) axis and sympathetic nervous system in stress response, as well as HPA alterations in PTSD [2,3,55]. Notwithstanding some discrepancies between observational studies [56,57], a meta-analysis found that morning and 24 h cortisol were significantly lower in PTSD than in controls [58]. In parallel, according to a systematic review that assessed the influence of light wavelength on human HPA axis rhythms, exposure to bright lights with stronger short-wavelength (blue/green) components in the early morning induced greater increases in cortisol compared to lights with stronger long-wavelength (red) components [59]. The reported variable effects of LT on other parameters, notably sleep and mood, might be explained by differences in the intensity and duration of exposure [22,26,60].

### 4.2. Strengths and Limitations of the Review

This systematic review is the first to address the effect of LT in patients with PTSD, and provide interesting results in terms of PTSD severity, but also symptoms related to sleep–wake disturbances, depression, and anxiety. This review also provides data about the impact of LT on the retention of fear extinction memory, and neuroimaging parameters. However, our results should be cautiously interpreted considering some limits.

The limitations of our review include the low number of included studies, which affects the generalization of our findings, as well as the fact that all studies were conducted in the USA. Also, most of the selected studies were conducted on a small number of participants, which limits the statistical power of these individual studies. Considering the changes in PTSD criteria from one DSM version to another, the inconsistency of PTSD diagnostic tool across studies can also be considered a limitation. Light therapy protocols across studies were heterogeneous in terms of duration and intensity, and the varied assessed outcomes (symptom severity and sleep and mood parameters) limited our ability to conduct meta-analyses. As for other limitations, the risk of bias was categorized as “some concern” in two studies, little was known about the period of the year when LT was applied (since seasons can influence the sensitivity to photic exposure), there was no long-term follow-up, and detailed information regarding the safety profile of the interventions was not reported in most included studies.

### 4.3. Implications of the Results for Practice, Policy, and Future Research

This literature synthesis provides promising results regarding the use of LT in PTSD, but these need to be strengthened with additional research. Indeed, it calls for further studies with some methodological adjustments such as larger sample sizes, a longer follow-up (6 or 12 months, for instance), a systematic use of updated PTSD diagnostic criteria, and the harmonization of LT parameters and outcomes (with, for instance, the use of a common tool to assess PTSD severity, sleep quality, depression, and anxiety symptoms). Also, it could be of interest to conduct trials in other countries, on patients with residual symptoms after psychotherapeutic or pharmacological interventions, or in combination with currently used PTSD treatments.

## 5. Conclusions

This study aims to synthesize the published knowledge on the effect of LT in patients with PTSD. LT is a therapeutic option that is relatively easy to implement, and with good patient acceptance. Our review found that the scarce studies on the topic report promising findings concerning PTSD symptoms, but inconsistent outcomes for the other parameters. Hence, further studies are needed for a more robust exploration of this research question, with methodological adjustments regarding the homogenization of PTSD diagnostic procedure, LT protocols, and assessed outcomes.

## Figures and Tables

**Table 1:** Eligibility criteria.

Inclusion Criteria	Exclusion Criteria
> Interventional studies on light therapy > Studies targeting patients with posttraumatic stress disorder > Studies assessing mental health (sleep–wake cycles, mood, anxiety, post-traumatic symptomatology, and extinction memory), neuroanatomical, or neurofunctional outcomes	> Studies on paediatric populations > Studies on animal models > Article types such as case reports, letters to the editor, comments, and reviews > Duplicated articles (especially the least recent duplicate) > Full-text unavailability > Studies in other languages other than English of French

**Table 2:** General characteristics of included studies.

Study	Location and Period	Design	Population (with PTSD Screening Tool)	Interventions in Each Arm [LT Group and Control Group if Applicable]	Sample Size and Male/Female Ratio	Mean Age (SD) and Age Range
Elliott et al. 2022 [32]	United States of America, August 2017 to August 2018	Uncontrolled Clinical Trial Open-label	Veterans with history of TBI, and having comorbid PTSD [assessed with the PTSD checklist for DSM-5 (PCL-5)]	Morning bright light therapy = lightbox (~10,000 lux at the eye) for 60 min every morning for 4 weeks (UCT)	18 and NR	NR
Killgore et al. 2022 [33]	United States of America, NR.	Randomized Controlled Trial Double-blind	Individuals meeting DSM-5 criteria for PTSD	Blue-light exposure treatment (BLT, peaking at λ = 469 nm, at 214 lux) versus a matched amber light treatment (ALT, peaking at λ = 578 nm, at 188 lux) [daily for 30 min over 6 weeks]	76 (39 on BLT versus 37 on ALT) and 25/51 (14/25 versus 11/26)	31.4 (8.8), from 20 to 49 years of age
Vanuk et al. 2022 [38]	United States of America, NR.	Randomized Controlled Trial Double-blind	Individuals with PTSD undergoing a well-validated fear conditioning/extinction protocol with assignment to interventions	Morning BLUE (BLT, peaking at λ = 469 nm, at 214 lux) or placebo AMBER (ALT, peaking at λ = 578 nm, at 188 lux) light therapy daily for 30 min over 6 weeks	82 (43 on BLT versus 39 on ALT) and 14/29 versus 12/27	31.7 (8.8) for BLT versus 30.2 (8.7) for ALT
Youngstedt et al. 2022 [39]	United States of America, NR.	Randomized Controlled Trial Single-blind	Veterans with PTSD attributable to combat in Afghanistan and/or Iraq (DSM-IV)	4 weeks of daily morning bright light treatment (10,000 lux for 30 min/day) or a control treatment (inactivated negative ion generator)	69 (34 for bright light and 35 for the control treatment)	NR
Zalta et al. 2019 [40]	United States of America, NR	Randomized Controlled Trial	Individuals with probable PTSD established using a self-report measure (however, participants had to have a PTSD Checklist for DSM-5 score > 33)	Active Re-timer^®^ † (*n* = 9) [(~500 nm, 500 lux)] versus placebo Re-timer^®^ dimmed with neutral density filters (*n* = 6), during one hour in the morning, for 4 weeks	15 (9 on the active treatment versus 6 on the placebo treatment), 7/8 (5/9 versus 2/6)	44.9 (11.8) [40.8 (9.3) versus 51 (13.3)]

LT = Light Therapy; TBI = Traumatic Brain Injury; PTSD = Post-Traumatic Stress Disorder; NR = Not Reported; DSM = Diagnostic and Statistical Manual for Mental Disorders. † Designed and manufactured by Flinders University in Australia.

**Table 3:** Risk of bias (ROB) assessment using the revised Cochrane risk-of-bias tool for randomized trials (RoB 2).

Study	D1: ROB Arising from the Randomization Process	D2a: ROB Due to Deviations from the Intended Interventions/Effect of Assignment to Intervention	D2b: ROB Due to Deviations from the Intended Interventions/Effect of Adherence to Intervention	D3: ROB Due to Missing Outcome Data	D4: ROB in Measurement of the Outcome	D5: ROB in Selection of the Reported Result	Overall ROB
Killgore et al. 2022 [33]	Low	Low	Low	Low	Low	Low	Low
Vanuk et al. 2022 [38]	Low	Some concern	Low	Low	Low	Low	Some concern
Youngstedt et al. 2022 [39]	Low	Some concern	Low	Low	Low	Low	Some concern
Zalta et al. 2019 [40]	Low	Low	Low	Low	Low	Low	Low

**Table 4:** Main results for each included study.

Study	Outcomes of Interest and Risk of Bias	Effects on Sleep Parameters	Effects on Other Mental Health Parameters
Elliott et al. 2022 [32]	> Sleep quality (with the ISI), TBI (with the NSI), PTSD (with the PCL-5), Mood (with the PHQ-9), Pain, Quality of life, sleep actigraphy parameters [total sleep time (TST), time in bed (TIB), sleep onset latency (SOL), sleep efficiency (SE), wake after sleep onset (WASO), total activity, average activity/epoch, and number of nocturnal awakenings] > Risk of bias = Low	> Subjects with comorbid PTSD had higher pre-intervention ISI scores: ISI with PTSD = 17.1±5.3, versus ISI without PTSD = 11.9±5.5. > Subjects with comorbid PTSD had a greater percent change post intervention: 15.1±16.9% with PTSD, versus 8.7±13.8% without PTSD. This corresponded to post-intervention ISI scores of 12.9±4.5 (TBI with PTSD), versus 9.5±4.6 (TBI without PTSD). > Considering a 4-point decrease in ISI score to define response to light therapy, subjects with comorbid PTSD had 5.76 times the odds (CI: 1.18–28.28; p = 0.041) of showing a response to light therapy compared to subjects without PTSD. > Based on these raw scores, subjects with PTSD shifted from “moderate” insomnia to “mild” insomnia, while those without PTSD remained in the “mild” category.	> Improvement of PTSD symptom severity. > No other specific results for patients with TBI and PTSD, regarding the other mental health parameters. > There were no documented important harms or unintended effects during the study.
Killgore et al. 2022 [33]	> Sleep outcomes assessed with questionnaires [Pittsburgh Sleep Quality Index (PSQI), Disturbing Dreams and Nightmares Severity Index (DDNSI), Insomnia Severity Index (ISI)] or by wrist actigraphy [time in bed (TIB), total sleep time (TST), sleep onset latency (SOL), wake after sleep onset (WASO), sleep efficiency (SE)] > Neuroimaging scans / neuroimaging data [analysed using the Computational Anatomy Toolbox (CAT12) and Voxel-Based Morphometry (VBM) modules] > Clinician-Administered PTSD Scale for DSM-5 (CAPS-5) > Risk of bias = Low	> The BLT condition produced significant increases in total time in bed (per-protocol and intention-to-treat analyses) and total sleep time from actigraphy compared to the ALT condition (only per-protocol analysis). > WASO and SE declined significantly for the ALT group but did not change for the BLT group (per-protocol and intention-to-treat analyses). > Light therapy did not significantly influence changes in PSQI total scores, in DDNSI scores, in ISI Total scores, or in SOL.	> BLT led to a significant increase in GMV within the left amygdala, compared to ALT, but did not affect hypothesized medial prefrontal regions (the expected increase in GMV within the medial prefrontal cortex was not found). > Within-group correlations showed that improvements in sleep quality and nightmare severity were correlated with increases in left amygdala volume over the course of treatment for the BLT group but not the ALT group.
Vanuk et al. 2022 [38]	> Epworth Sleepiness Scale (ESS), Pittsburgh Sleep Quality Index (PSQI), Functional Outcomes of Sleep Questionnaire (FOSQ), Insomnia Severity Index (ISI), Disturbing Dreams and Nightmares Severity Index (DDNSI) > Clinician-Administered PTSD Scale for DSM-5 (CAPS-5), Post-traumatic Stress Disorder Checklist for DSM-5 (PCL-5) > Skin conductance response (during fear conditioning, extinction learning), electrocardiogram, brain activation through functional magnetic resonance imaging (fMRI), anatomical neuroimaging data > Risk of bias = Some concern	> Individuals in both the ALT and BLT reported improved sleep as measured by lower PSQI scores (indicating fewer symptoms of disrupted sleep) and higher FOSQ scores. > Both groups also reported lower levels of insomnia severity as measured by the ISI and nightmares as measured by the DDNSI. > Changes in daytime sleepiness as measured by the ESS were not significant. To summarize, subjective sleep tended to improve between baseline and post-treatment. However, this improvement was not qualified by significant interaction effects and both groups tended to show similar improvements.	> With LT, there was a strong effect of time on PTSD severity, beta = −0.62, 95% CI [−0.80, −0.44], t(154) = −6.67, *p* < 0.001, as assessed by the CAPS-5. There was a decrease in PTSD symptoms, as assessed by the PCL-5, beta = −0.38, 95% CI [−0.52, −0.24], t(155) = −5.24, *p* < 0.001. Nevertheless, both groups improved in PTSD symptoms and severity (no significant differences between the active and the placebo group). > Participants receiving BLT also sustained retention of the extinction memory, while those in the placebo amber light treatment group showed impairment, characterized by the restoration of the extinguished fear response after 6 weeks. > Daily BLUE-wavelength morning light exposure was associated with greater retention of extinction learning in patients with PTSD when compared to ALT. > Participants in the ALT also demonstrated greater reactivity in the left insula when viewing the previously extinguished fear-conditioned stimuli in a novel context. > BLT resulted in a significant decrease in activation responses within the left insular cortex relative to ALT. In all, BLT promotes the consolidation of extinction memories via improved sleep. > Sex differences in extinction recall.
Youngstedt et al. 2022 [39]	> Pittsburgh Sleep Quality Index (PSQI) and PSQI Addendum for PTSD (PSQIADD). > Sleep and the circadian rhythm parameters assessed through wrist actigraphy. > Clinician-Assessed PTSD Scale (CAPS), Clinical Global Impressions Scale (CGI), Hamilton Depression Scale, PTSD Checklist-Military (PCL-M) with a self-rating, Hamilton Depression Scale, Hamilton Atypical Symptoms, Clinical Global Impressions (CGI) scale. > Self-reported scales of state anxiety [State-Trait Anxiety Inventory (STAI Form Y-2)], depression [Beck Depression Inventory (BDI)], and side effects [Systematic Assessment for Treatment Emergent Effects (SAFTEE) side effect questionnaire] > Risk of bias = Some concern	> Changes in actigraphic estimates of sleep (TST, WASO, and SE) and changes in PSQI and PSQI Addendum items did not differ significantly between treatments. > Of note, the slope differences [95% confidence interval] (control vs. bright light) for PSQI, PSQI Addendum, TST, WASO, and SE, were, respectively, −0.2 [−0.6; 0.2], −0.2 [−0.6; 0.2], 3.4 [−6.4; 13.2], 0.2 [ −0.5; 0.9], and 1.4 [−1.0; 3.7].	> Compared with the control treatment, bright light elicited significantly greater improvements in the CAPS (Intergroup difference in before—after variation = −9.52 with a 95% confidence interval from −18.48 to −0.55) and the CGI. There was a significantly better CGI-IM score following the bright light treatment compared with the control treatment (coefficient: −0.57, P=0.034). > Improvement. The bright light also elicited a significantly greater rate of treatment response (reduction ≥33%) for the CAPS (44.1% vs. 8.6%) and PCL-M (33% vs. 6%), but no participant had remission from PTSD. > The bright light elicited a phase advance of the activity acrophase, whereas the control treatment resulted in a phase delay, with a statistically significant difference. > Changes in depression (Hamilton scales) and anxiety did not differ between treatments. Improvement in CAPS was significantly correlated with a phase advance of the circadian rhythm of wrist activity. > There was a lack of significant treatment difference in PCL-M, BDI, STAI, and PSQI at 1 month, 4 months, and 8 months (data, respectively, obtained from 29, 24, and 16 participants). > Regarding safety, the number of symptoms endorsed and number of symptoms pertaining to the head, eyes, and mania/agitation decreased following both treatments. There was no significant treatment effect for any of these metrics.
Zalta et al. 2019 [40]	> PCL-5, PHQ-9, PSQI. > Minimal clinically important difference (MCID) for the DSM-IV version of the PTSD checklist. This MCID is defined as a 10-point improvement. > Objective actigraphy estimates of sleep onset time, wake time, total sleep time, and wake after sleep onset. > Risk of bias = Low	Active group participants reported greater improvements in subjective sleep quality from pre- to post-treatment than placebo participants. Also, they saw a greater advance in wake time relative to placebo participants, a decrease in TST from pre- to post-treatment of approximately 36 min, and a decrease in WASO from pre- to post-treatment (whereas placebo participants showed a slight increase in WASO from pre- to post-treatment). However, there were no statistical differences regarding all sleep parameters.	> Those in the active group were more likely to achieve a minimal clinically important change in PTSD and depression symptoms than those in the placebo group. > Participants in the active group also had larger reductions in PTSD and depression symptoms from pre- to post-treatment than those in the placebo group (*d* = 0.94 and 0.74, respectively). > The treatment was also well tolerated by participants.

LT = Light Therapy; ISI = Insomnia Severity Index; PHQ = Patient Health Questionnaire; NSI = Neurobehavioral Symptom Inventory.

## Supplementary Materials

The following supporting information can be downloaded at: https://www.mdpi.com/article/10.3390/jcm13133926/s1, Table S1: PRISMA checklist. Table S2: PICO framework applied to our review. Table S3: Search strategies. Reference [34] is cited in the Supplementary Materials.
